# RATIO OF METASTATIC LYMPH NODES VS. RESECTED LYMPH NODES (N-RATIO) HAS PROGNOSTIC IMPLICATIONS IN GASTRIC CANCER

**DOI:** 10.1590/0102-6720202400031e1824

**Published:** 2024-09-23

**Authors:** Breno Cordeiro PORTO, Marina Alessandra PEREIRA, Marcus Fernando Kodama Pertille RAMOS, André Roncon DIAS, Fábio Pinatel LOPASSO, Luiz Augusto Carneiro D’ALBUQUERQUE, Ulysses RIBEIRO

**Affiliations:** 1 Universidade de São Paulo, School of Medicine, Cancer Institute of the State of São Paulo – São Paulo (SP), Brazil.

**Keywords:** Stomach Neoplasms, Adenocarcinoma, Neoplasm Metastasis, Neoplasm Staging, Neoplasias Gástricas, Adenocarcinoma, Metástase Neoplásica, Estadiamento de Neoplasias

## Abstract

**BACKGROUND:**

Lymph node status is vital for gastric cancer (GC) prognosis, but the conventional pN stage may be limited by variations in lymphadenectomy and stage migration. The N-Ratio, which assesses the ratio of metastatic to resected lymph nodes, emerges as a promising prognostic tool.

**AIMS:**

To assess N-Ratios prognostic value in GC, particularly in patients with <25 resected lymph nodes.

**METHODS:**

Patients who underwent gastrectomy with curative intent for GC were retrospectively evaluated. The N-Ratio categories were determined using the ROC curve method, and the area under the curve (AUC) was used as a measure of performance in predicting recurrence/death.

**RESULTS:**

A total of 561 GC patients were included in the study, 57% had pN+ status, and 17.5% had <25 resected lymph nodes. N-Ratio, with a mean of 0.12, predicted survival with 74% accuracy (AUC=0.74; 95%CI 0.70–0.78, p<0.001). N-Ratio categories included: N-Ratio 0 (43%); N-Ratio 1 (12.3%); N-Ratio 2 (31.6%); and N-Ratio 3 (13.2%). Disease-free survival (DFS) varied among all N-Ratio groups, with N-Ratio 3 showing worse survival than pN3 cases (DFS=21.8 vs. 11 months, p=0.022, p<0.05). In cases with <25 resected lymph nodes, DFS was not significantly worse in N-Ratio 0 (68.8 vs. 81.9%, p=0.061, p>0.05) and N-Ratio 1 (66.2 vs. 50%, p=0.504, p>0.05) groups. The DFS of N-Ratio-0 cases with <25 lymph nodes was similar to N-Ratio 1 cases.

**CONCLUSIONS:**

N-Ratio influenced survival in GC patients, especially in advanced lymph node disease (N-Ratio 3). Considering that N-Ratio does not impact pN0 cases, individualized prognosis assessment is essential for patients with <25 resected lymph nodes.

## RESUMO

**RACIONAL:**

O status linfonodal é vital para o prognóstico do câncer gástrico (CG), mas o estágio pN convencional pode ser limitado por variações na linfadenectomia e migração do estágio. O N-Ratio, que avalia a proporção entre linfonodos metastáticos e linfonodos ressecados, surge como uma ferramenta prognóstica promissora.

**OBJETIVOS:**

Avaliar o valor prognóstico do N-Ratio no CG, particularmente em pacientes com <25 linfonodos ressecados.

**MÉTODOS:**

Foram analisados, retrospectivamente, pacientes submetidos à gastrectomia com intenção curativa para CG. As categorias do N-Ratio foram determinadas pelo método da curva ROC, e a área sob a curva (AUC) foi utilizada como medida de desempenho na previsão de recorrência/óbito.

**RESULTADOS:**

Foram incluídos no estudo 561 pacientes com CG, 57% tinham status pN+ e 17,5% tinham <25 linfonodos ressecados. O N-Ratio, com média de 0,12, previu a sobrevida com precisão de 74% (AUC=0,74; IC95% 0,70–0,78, p<0,001). As categorias N-Ratio incluíram: N-Ratio 0 (43%); N-Ratio 1 (12,3%); N-Ratio 2 (31,6%); e N-Ratio 3 (13,2%). A sobrevida livre de doença (SLD) variou entre todos os grupos N-Ratio, com o N-Ratio 3 apresentando pior sobrevida do que os casos pN3 (SLD=21,8 vs.11 meses, p=0,022, p<0,05). Nos casos com <25 linfonodos ressecados, a SLD não foi significativamente pior nos grupos N-Ratio 0 (68,8 vs. 81,9%, p=0,061, p>0,05) e N-Ratio 1 (66,2 vs. 50%, p=0,504, p>0,05). A SLD dos casos de N-Ratio 0 com <25 linfonodos foi semelhante aos casos de N-Ratio 1.

**CONCLUSÕES:**

O N-Ratio influenciou a sobrevida em pacientes com CG, especialmente na doença linfonodal avançada (N-Ratio 3). Considerando que a proporção N não afeta os casos pN0, a avaliação prognóstica individualizada é essencial para pacientes com <25 linfonodos ressecados.

## INTRODUCTION

Gastric cancer (GC) is one of the most common neoplasms worldwide, ranking fifth in incidence and fourth in mortality among all cancers^9,28^.

Lymph node status is crucial for determining the prognosis of patients with GC, particularly in those with more advanced cancers, in whom metastasis may occur more frequently. In this regard, gastrectomy with D2 lymphadenectomy is the gold-standard treatment for patients with locally advanced GC^2,6,14^.

In GC, lymphatic dissemination is more prevalent than hematogenous spread, which justifies lymphadenectomy during surgery^5^. Lymphadenectomy can be D1, in which the lymph nodes closest to the stomach are resected, or D2, in which the resection is expanded through the vessels that supply the stomach^27^. D2 lymphadenectomy is associated with better survival^25,27^, although it is associated with higher rates of postoperative complications especially in older, high-risk patients with comorbidities^14,23,26^.

Despite the reliability and simplicity of the TNM classification, it has some issues related to the correct number of lymph nodes (LNs) to be resected for an accurate staging. The Union for International Cancer Control/American Joint Committee on Cancer (UICC/AJCC) recommends that at least 15 LNs could be examined for correct staging, while the Japanese Gastric Cancer Association (JGCA) recommends that more than 25 lymph nodes is ideal for correct staging^11,12,17^. Some studies also suggest that this classification is involved in problems with stage migration, related to the Will Rogers Phenomenon^3,8^, which can lead to some patients being incorrectly staged, as the patient’s stage may vary due to lymph node involvement^4,5,29^.

In order to reduce stage migration, some investigators have proposed using the N-Ratio, namely the ratio between metastatic LNs and the total number of LNs examined, as a new prognostic indicator for GC, even in the case of limited LN dissection^7,18,21,31^.

Although some researchers have reported that N-Ratio is an independent prognostic factor, to date there is no N-Ratio system considered standard for use^13,15,20,21,30^. Different N-Ratio systems with different cutoff values had been proposed^20,29,31^, and there are controversies as to whether its applicability would be restricted to cases with LN yield, patients after neoadjuvant chemotherapy (CMT), or remnant gastric cancer^21,24,25^.

Thus, the aim of this study was to investigate the prognostic value of N-Ratio in patients with GC after curative surgical treatment. We also evaluated the influence on survival in patients with fewer than 25 resected LNs.

## METHODS

Patients with GC who underwent surgical procedures at our Institution between 2009 and 2020 were evaluated from our prospectively collected database.

Patients were selected for the study according to the following eligibility criteria:

1. Histological diagnosis of gastric adenocarcinoma;
2. Total or subtotal gastrectomy, with D1 or D2 lymphadenectomy;
3. At least two years of follow-up; and
4. Surgery with curative intent. Patients with remnant gastric cancer, 90-day mortality, presence of synchronous or metachronous neoplasia, and palliative or emergency surgeries were excluded.

All patients underwent gastrectomy with lymphadenectomy according to the guidelines of the JGCA^4^. The eighth edition of the TNM classification was used for staging.

The surgical specimens were evaluated by histopathological criteria according to the College of American Pathologists protocol (CAP — Cancer Protocols and Checklists), as already performed in our routine.

The clinical data evaluated included sex, age, Body Mass Index (BMI), hemoglobin levels, albumin levels, neutrophil-to-lymphocyte ratio (NLR), American Society of Anesthesiologists (ASA) classification, Charlson Comorbidity Index (CCI), type of gastrectomy, extension of lymphadenectomy, and CMT.

The follow-up was performed after surgery every three months in the first year and every six months in subsequent years. This study was approved by the hospital’s Ethics and Research Committee and is registered at *Plataforma Brasil* (a national and unified database of records and research involving human beings) (CAEE: 54787422.3.0000.0068).

### N-Ratio classification

The N-Ratio was calculated for each patient according to the following formula: N-Ratio=number of positive lymph nodes/number of resected lymph nodes.

The area under the receiver operating characteristic (ROC) curve (AUC) was used to evaluate the metric performance of the N-Ratio in predicting recurrence or death. The optimal cutoff value was determined by maximizing Youden’s index (sensitivity + specificity - 1) to distinguish N-Ratio 1 and N-Ratio 2. To define the N-Ratio category 3, the cutoff value was obtained in which the specificity reached 70%. The predictive capacity of the N-Ratio categories was tested by Kaplan-Meier survival method analysis.

### Statistical analysis

Data were described using the mean (with SD, standard deviation) and median (with IQR, interquartile range) quantitative variables, and frequency for qualitative variables.

The differences between the groups were analyzed using the chi-square test for nominal variables and t-test, ANOVA, or Kruskal-Wallis test for continuous variables.

Survival was estimated by the Kaplan-Meier method, and the log-rank test was used to identify statistical differences between groups. Disease-free survival (DFS) was calculated from the date of surgery until the date of recurrence or death from any cause. Alive patients were censored at the date of the last follow-up. The Cox proportional hazards model was used to identify risk factors related to survival. Significant variables in the univariate analysis were included in the multivariate model to verify those independently associated with survival outcomes. The results were reported as hazard ratios (HRs) with 95% confidence intervals (CI).

All analyses were performed using SPSS version 20.0 (SPSS, Chicago, IL, USA). Results were considered statistically significant when p<0.05.

## RESULTS

A total of 561 patients who met the eligibility criteria were included in the study. The clinicopathological and surgical data for these patients are summarized in Table 1.

**Table 1:** Clinicopathologic and surgical characteristics of 561 patients with gastric cancer.

Variables	n=561	%
Sex		
Women	239	42.6
Men	322	57.4
Age (years)		
Mean (SD)	61.9 (12.6)	
Min.–Max.	22.7–94.5	
BMI (Kg/m^2^)		
Mean (SD)	24.6 (4.5)	
Hemoglobin (g/dL)		
Mean (SD)	12.3 (2.1)	
Albumin (g/dL)		
Mean (SD)	4.0 (1.5)	
Neutrophil-to-lymphocyte ratio		
Mean (SD)	2.7 (2.7)	
American Society of Anesthesiologists		
I/II	434	77.4
III/IV	127	22.6
Charlson Comorbidity Index*		
0	374	66.7
≥1	187	33.3
Type of gastrectomy		
Subtotal	358	63.8
Total	203	36.2
Lymphadenectomy		
D1	81	14.4
D2	480	85.6
Tumor size (cm)		
Mean (SD)	4.8 (3.0)	
Lauren type		
Intestinal/Indeterminate	309	55.1
Diffuse/Mixed	252	44.9
Histological differentiation		
Well/moderately differentiated	259	46.2
Poorly differentiated	302	53.8
Lymphatic invasion		
No	287	51.2
Yes	274	48.8
Venous invasion		
No	376	67.0
Yes	185	33.0
Perineural invasion		
No	292	52.0
Yes	269	48.0
T status		
pT1	155	27.6
pT2	71	12.7
pT3	179	31.9
pT4	156	27.8
pN status		
pN0	241	43.0
pN1	80	14.3
pN2	102	18.2
pN3	138	24.6
No. of resected lymph nodes		
Mean (SD)	41.2 (18.2)	
Total of Lymph nodes		
LNs <25	98	17.5
LNs ≥25	463	82.5
pTNM		
I	182	32.4
II	128	22.8
III	251	44.7

SD: standard deviation; BMI: Body Mass Index; T: Tumor; LN: lymph node; TNM: tumor staging.

The mean age of the patients was 61.9 years (range 22.7–94.5 years), with 322 men (57.4%) and 239 women (42.6%).

The median number of resected LNs was 41.2 (range 4–115), and 57% of the patients had lymph nodal metastasis. In addition, 17.5% of patients had fewer than 25 resected LNs. Most cases were staged as pTNM III (44.7%), and 56.7% of patients received preoperative or adjuvant chemotherapy (91 and 273 cases, respectively).

**Figure 1:**
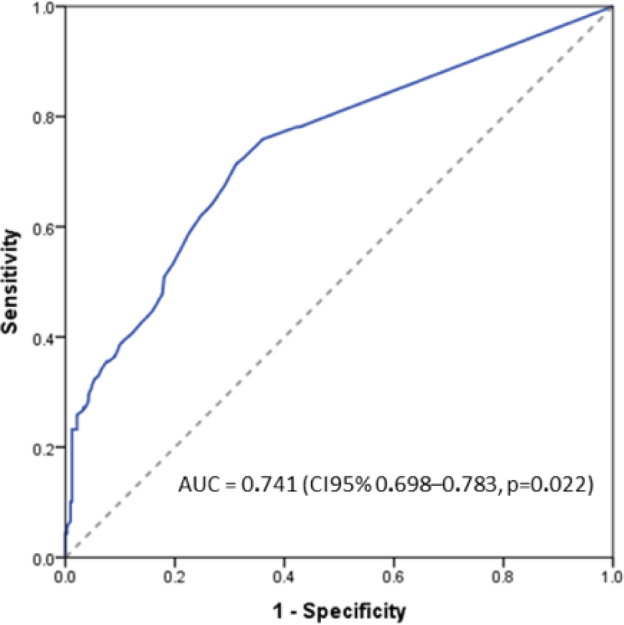
Receiver operating characteristic curve and the area under the curve (AUC) for N-Ratio.

The N-Ratio was calculated for each patient, and the median value was 0.12. The accuracy of N-Ratio in predicting DFS was evaluated using a ROC curve, and the AUC was used to determine its accuracy, as presented in Figure 1. The N-Ratio had an accuracy of 74% (AUC=0.741, 95%CI 0.698–0.783, p=0.022). Based on sensitivity and specificity values, the patients were classified into the following N-Ratio groups using the cutoff values: N-Ratio 0: 0%; N-Ratio 1: 0–5%; N-Ratio 2: 6–33%; N-Ratio 3: >34%. The established cutoff values and the distribution of patients in the N-Ratio categories are presented in Table 2.

AUC: area under the curve.

**Table 2:** Cutoff values and distribution of 561 patients in N-Ratio groups.

N-Ratio	Cutoff values	n=561	%
N-Ratio 0	0	241	43.0
N-Ratio 1	0.01–0.05	69	12.3
N-Ratio 2	0.06–0.33	177	31.6
N-Ratio 3	>0.34	74	13.2

Clinicopathological characteristics in relation to the N-Ratio groups are presented in Table 3. The frequency of total gastrectomy and D1 lymphadenectomy was higher in the N-Ratio 3 group. Furthermore, larger tumor size, diffuse histological type, poorly differentiated histology, and advanced pT and pTNM stage were also more common in the N-Ratio 3 category. Lower frequency of lymphatic, venous, and perineural invasion were observed in the N-Ratio 0 category.

**Table 3:** Clinicopathological characteristics of gastric cancer of 561 patients according to the N-Ratio categories.

Variables	N-Ratio 0	N-Ratio 1	N-Ratio 2	N-Ratio 3	p
	n=241 (%)	n=69 (%)	n=177 (%)	n=74 (%)	
Sex					
Women	111 (46.1)	32 (46.4)	62 (35.0)	34 (45.9)	0.108\
Men	130 (53.9)	37 (53.6)	115 (64.9)	40 (54.1)	
Age (years)					
Mean (SD)	62.7 (12.7)	60.3 (12.6)	62.6 (12.3)	59.1 (12.7)	0.094
Type of gastrectomy					
Subtotal	173 (71.8)	42 (60.9)	109 (61.6)	34 (45.9)	0.001
Total	68 (28.2)	27 (39.1)	68 (38.4)	40 (54.1)	
Type of Lymphadenectomy					
D1	42 (17.4)	5 (7.2)	19 (10.7)	15 (20.3)	0.035
D2	199 (82.6)	64 (92.8)	158 (89.3)	59 (79.7)	
Tumor size (cm)					
Mean	3.9 (2.8)	4.9 (2.8)	5.4 (2.7)	6.1 (3.0)	<0.001
Lauren type					
Intestinal	154 (63.9)	43 (62.3)	88 (49.7)	24 (32.4)	<0.001
Diffuse/Mixed	87 (36.1)	26 (37.7)	89 (50.3)	50 (67.6)	
Histological differentiation					
Well/moderately differentiated	134 (55.6)	34 (49.2)	74 (41.8)	17 (22.9)	<0.001
Poorly differentiated	107 (44.3)	35 (50.7)	103 (58.1)	57 (77)	
Lymphatic invasion					
No	187 (77.6)	35 (50.7)	53 (29.9)	12 (16.2)	<0.001
Yes	54 (22.4)	34 (49.3)	124 (70.1)	62 (83.8)	
Venous invasion					
No	208 (86.3)	49 (71.0)	90 (50.8)	29 (39.2)	<0.001
Yes	33 (13.7)	20 (29.0)	87 (49.2)	45 (60.8)	
Perineural invasion					
No	190 (78.8)	33 (47.8)	53 (29.9)	16 (21.6)	<0.001
Yes	51 (21.2)	36 (52.2)	124 (70.1)	58 (78.4)	
T status					
pT1/pT2	166 (68.9)	27 (39.1)	30 (16.9)	3 (4.1)	<0.001
pT3/pT4	75 (31.1)	42 (60.9)	147 (83.1)	71 (95.9)	
pN status					
pN0	241 (100)	0 (0)	0 (0)	0 (0)	–
pN1	0 (0)	63 (91.3)	17 (9.6)	0 (0)	
pN2	0 (0)	6 (8.7)	93 (52.5)	3 (4.1)	
pN3	0 (0)	0 (0)	67 (37.9)	71 (95.9)	
No. of resected lymph nodes					
Mean (SD)	38.8 (17.9)	46.2 (15.3)	43.4 (19.0)	39.2 (18.8)	0.005
Total of lymph nodes					
LNs <25	48 (19.9)	4 (5.8)	27 (15.3)	19 (25.7)	0.009
LNs ≥25	193 (80.1)	65 (94.2)	150 (84.7)	55 (74.3)	
pTNM					
I/II	236 (97.9)	67 (97.1)	29 (16.4)	0 (0)	<0.001
III	5 (2.1)	2 (2.9)	148 (83.6)	74 (100)	

SD: standard deviation; T: Tumor; LN: lymph node; TNM: tumor staging.

The groups also differed regarding the mean number of resected lymph nodes, with the highest frequency of cases with fewer than 25 LNs in the N-Ratio 3 group (25.7% of cases). The N-Ratio 1 category presented the highest frequency of cases, with at least 25 resected LNs (94.2% of cases).

### Survival analysis

The median follow-up for the entire cohort was 47.1 months (mean = 48.2). During the follow-up, 160 (28.5%) patients had disease recurrence, and 204 (36.4%) died. The estimated 5-year DFS rate for the entire cohort was 57.4%.

**Figure 2:**
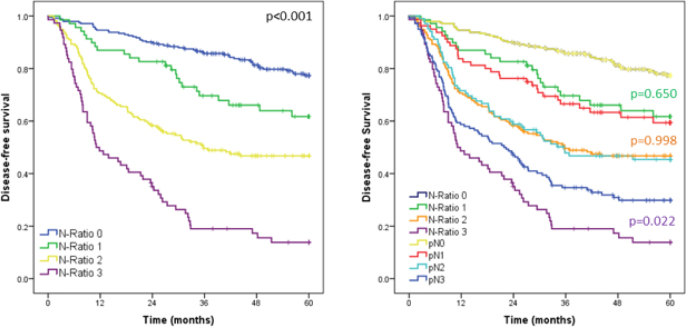
Survival curves of 561 patients according to the N-Ratio categories and comparison of the N-Ratio groups with the pN stage.

The curves of all N-Ratio categories differed from each other (Figure 2). N-Ratio-0 patients had better survival compared to N-Ratio 1 (p=0.005); while N-Ratio-1 cases had better survival compared to patients classified as N-Ratio 2 (p=0.007). In turn, N-Ratio-3 GC had poorer survival compared to the N-Ratio 2 group (p<0.001).

When compared to the classic staging pN stage, there was no statistical difference between the DFS of N-Ratio 1 and pN1 group (p=0.650), and between N-Ratio 2 and pN2 group (35.8 vs 35.7 months, respectively; p=0.998). Nevertheless, the DFS in the N-Ratio 3 category was worse compared to pN3 group (p=0.022). The median survival of the N-Ratio 3 and pN3 was 11.1 and 21.8 months, respectively.

### Survival according to the number of resected lymph nodes: <25 and ≥25 LNs groups

**Figure 3:**
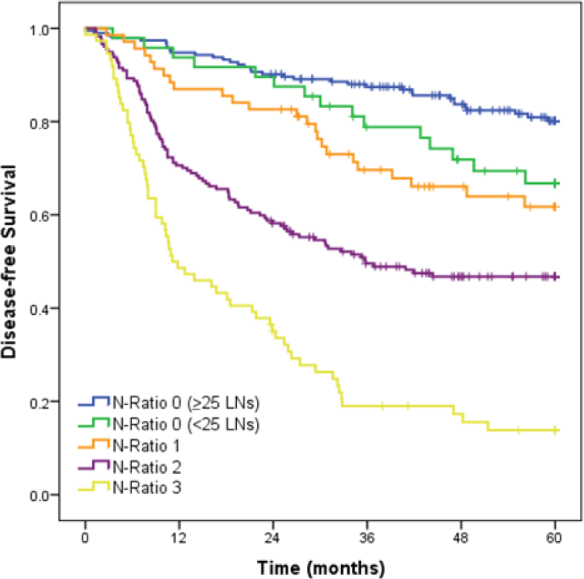
Survival curves of 561 patients comparing N-Ratio 0 <25 LNs vs ≥25 LNs.

Survival data for the N-Ratio and pN categories stratified by the number of LNs are shown in Table 4. There was no significant difference between categories when comparing cases with <25 LNs and those with ≥25 LNs. However, a tendency toward better survival was observed in N-Ratio-0/pN0 patients with ≥25 resected LNs compared to <25-LNs cases (Figure 3).

**Table 4:** Disease-free survival of 561 patients based on N-Ratio classification and N stage according to the number of resected lymph nodes.

N-Ratio	n	n of events	Median DFS (months)	p
Ratio 0				
<25 LNs	48	15	nr	0.061
≥25 LNs	193	35	nr	
Ratio 1				
<25 LNs	4	2	56.1	0.504
≥25 LNs	65	22	nr	
Ratio 2				
<25 LNs	27	14	26.4	0.896
≥25 LNs	150	78	35.8	
Ratio 3				
<25 LNs	19	14	12.7	0.185
≥25 LNs	55	48	10.6	
**N stage**	**n**	**n of events**	**Median DFS (months)**	**p**
N1				
<25 LNs	10	6	20.7	0.092
≥25 LNs	70	24	nr	
N2				
<25 LNs	24	13	26.4	0.935
≥25 LNs	78	41	35.7	
N3				
<25 LNs	16	11	12.7	0.908
≥25 LNs	122	83	21.8	

nr: not reached; DFS: disease-free survival; LN: lymph node.

Survival data for cases with <25 and ≥25 LNs, comparing the N-Ratio and pN categories with each other, are presented in Table 1.

In the group with <25 LNs, N-Ratio 1 presented higher DFS median compared to pN1 (56.1 vs 20.7 months, p=0.593), while the estimated 5-year DFS was worse in N-Ratio 3 compared to pN3 (22.2 vs 28.6 months, p=0.849). In the group with ≥25 LNs, the comparison of the estimated 5-year DFS of N-Ratio 3 and pN3 showed significant statistic values (11.0 vs. 30.0, p=0.021, p<0.05) (Table 5).

**Table 5:** Disease-free survival rates of 561 patients according to the number of resected lymph nodes for N-Ratio and pN stages.

Groups	N Category	n	n of events	Estimated 5-year DFS (%)	Median DFS	p
**<25 LNs**	N-Ratio 0/pN0	48	15	66.7	nr	–
	N-Ratio 1	4	2	–	56.1	0.593
	pN1	10	6	25	20.7	
	N-Ratio 2	27	14	48.1	26.4	0.880
	pN2	24	13	43.7	26.4	
	N-Ratio 3	19	14	22.2	12.7	0.849
	pN3	16	11	28.6	12.7	
**≥25 LNs**	N-Ratio 0/pN0	193	35	80.1	nr	–
	N-Ratio 1	65	22	63.5	nr	0.896
	pN1	70	24	63.2	nr	
	N-Ratio 2	150	78	46.9	35.8	0.951
	pN2	78	41	45.6	35.7	
	N-Ratio 3	55	48	11.0	10.6	0.021
	pN3	122	83	30.0	21.8	

nr: not reached; LN: Lymph node.

In the analysis of risk factors associated with DFS (Table 2), American Society of Anesthesiology (ASA) III/IV, total gastrectomy, pT3/T4 stage, and advanced N-Ratio category were independent factors associated with worse survival. Stepwise regression analysis included all the statistically significant prognostic factors by univariate analysis (Table 6).

**Table 6:** Univariate and multivariate analysis for disease-free survival of 561 gastric cancer patients.

Disease-free survival	Univariate			Multivariate		

Variables	HR	95%CI	p	HR	95%CI	p
Men (vs women)	1.19	0.91–1.55	0.198	–	–	–
Age >65 (vs <65 years)	1.06	0.81–1.37	0.685	–	–	–
ASA III/IV (vs ASA I/II)	1.53	1.15–2.05	0.004	1.36	1.02–1.82	0.038
Total gastrectomy (vs subtotal)	1.96	1.51–2.55	<0.001	1.67	1.28–2.17	<0.001
Diffuse/mixed (vs others)	1.49	1.15–1.93	0.003	1.17	0.89–1.53	0.255
<25 LNs (vs ≥25 LNs)	1.16	0.84–1.61	0.366	–	–	–
pT3/T4 (vs pT1/T2)	3.71	2.68–5.15	<0.001	2.02	1.39–2.93	<0.001
N-Ratio 0						
vs N-Ratio 1	1.90	1.17–3.10	0.010	1.41	0.86–2.34	0.177
vs N-Ratio 2	3.55	2.51–5.02	<0.001	2.41	1.65–3.53	<0.001
vs N-Ratio 3	8.36	5.72–12.20	<0.001	4.59	2.97–7.12	<0.001

HR: Hazard Ratio; LN: lymph node; ASA: American Society of Anesthesiology; CI: confidence interval.

## DISCUSSION

The present study was developed with the objective of evaluating the prognostic meaning of the N-Ratio classification in patients with GC treated with curative intent. N-Ratio can in fact be used as an alternative prognostic tool to better stage patients with GC. In addition, the prognostic value of N-Ratio based on the numbers of resected LNs (<25 or ≥25) was also evaluated.

We found that the categories determined by N-Ratio are related to independent factors associated with survival, which can lead to a better distinction between patients with a more extent lymph nodal stage and a more deteriorated prognosis. In addition, we could see a possibility to better stage N-Ratio-0 patients when stratified in groups with <25 resected LNs and ≥25 resected LNs.

N-Ratio has been indicated as an effective prognostic tool in several Western and Japanese series due to its capacity to better discriminate subsets of patients with similar prognosis and the ability to reduce the risk of stage migration^13,15,17,19-21,29-31^.

Some researchers only analyzed fragments of the population, as the study conducted by Jiang et al.^15^, who excluded patients with metastasis; for instance, Xu et al.^29^ and Bando et al.^1^ only considered D2 cases. Conversely, several other researchers^13,17,20,22,30,31^, including us, evaluated patients who underwent gastrectomies with curative intent associated with D1 and D2 lymphadenectomy. Furthermore, we could analyze in our study an average of 41.2 resected LNs, which is a noteworthy parameter considering that previous research reported values ranging from 10 to 51 LNs^1,7,13,15,17,20,22,30,31^.

Concerning GC, different N-Ratio cutoffs have been proposed. In an 1,853 multicentric study, Marchet et al.^20,21^ adopted the following N-Ratio cutoffs: 0%, 1 to 9%, 10 to 25%, and >25%, which was obtained based on log-rank test and the Martingale residual analysis. The same cutoffs of Marchet et al.^21^ were used in Xu et al.^29^ analysis, with 906 GC patients. Alternatively, in a study on 351 patients, Yamashita et al.^31^ selected four different N-Ratio cutoff values: 0%, 0 to 20%, 21 to 30%, and >30%, and the cutoff point survival analysis was the method used to determine these cutoffs. Based on the ROC curve and the AUC, we could evaluate the metric performance of the N-Ratio in predicting recurrence or death. Finally, we could propose the following cutoff values: 0%, 1 to 5%, 6 to 33%, and >34%. As we can observe, these values differ from those usually found in current literature and, therefore, can be seen as an alternative to future research.

Retrospective analyses of patients who underwent gastrectomies, such as those by Marchet et al.^20,21^ and Xu et al.^29^, have shown that the N-Ratio can be used as an independent prognostic factor in all cases, including those with few resected LNs (<15 LNs), when comparing survival to TNM staging. However, authors of other research, such as the one conducted by Mullaney et al.^22^, have suggested that the accuracy of N-Ratio in staging may be compromised when fewer than 15 LNs are removed during resection. This highlights the dilemma and emphasizes the need for future research to consider the amount of LNs to be resected carefully. In comparison with this other analysis, in this study we divided patients in those with <25 LNs and ≥25 LNs, as recommended by JGCA, seeking to find relevance and measure the applicability of N-Ratio even in less extensive LNs resections.

Indeed, this debate around the application of the N-ratio in relation to the average of recovered LNs is particularly important, as the TNM system does not consider lymph nodes that may have the potential to become cancerous, leading to inaccurate staging in cases with fewer resected structures.

Our findings underline this ability of N-Ratio to better stage patients with GC, mainly for those with a worse prognosis, when compared to the stage provided for pN, as we could see the results of median DFS of N-Ratio 3 vs. pN3 (DFS=11.1 vs. 21.8 months, p=0.022, p<0.05).

Taking this into consideration, this series not only corroborates the superiority of N-Ratio above the pN staging system as an alternative prognostic tool to better stage patients with GC, but also has a greater capacity to benefit patients with a more deteriorated prognosis, allowing a better treatment management^16^.

Interestingly, even with the difference between N-Ratio 3 and pN3, we observed no differences in survival between N-Ratio 1 vs pN1 and N-Ratio 2 and pN2 groups. This suggests that the N-Ratio was able to reclassify patients who would be understaged by the pN classification.

It should be noted that when submitted to statistical analysis, all the DFS curves of N-Ratio were different from each other (p<0.05), which reinforces the cutoffs previously proposed, ensuring statistical difference between all the N-Ratio groups evaluated. Besides, most of the research compared in this study also presented statistical difference between the survival curves^13,16,17,20,30,31^.

We divided the N-Ratio groups of our cohort into those with fewer than 25 LNs, or ≥25 resected LNs, as recommended by JGCA^14^. 17.5% of the patients presented fewer than 25 resected LNs, and while N-Ratio 1 had the greater number of resected lymph nodes (94.2% of patients ≥25 LNs), N-Ratio 3 presented the worst rate (74.3% of patients >25 LNs). This low amount of resected LNs is certainly linked to the more deteriorated prognosis of patients in the N-Ratio 3 group.

Noteworthily, we found significance difference in DFS in N-Ratio 0 when stratified by number of LNs (<25 vs. ≥25 LNs). Moreover, the N-Ratio 0 <25 LNs showed a survival comparable to the N-Ratio 1 (50.3 vs. 46.5 months, respectively), suggesting an indisputable worse prognosis of the group with fewer resected LNs. This observation may be relevant because the indication of adjuvant chemotherapy is based on the TNM stage. Thus, patients pN0 with fewer resected lymph nodes may be suitable to adjuvant chemotherapy.

Most importantly, these values imply that the N-Ratio 0 group was likely understaged and the patients classified as such may instead turn out to be N-Ratio 1 or N-Ratio 2. This raises concerns regarding the adequacy of LNs dissection in the N-Ratio 0 group, considering that, in cases in which there are no positive LNs, patients are automatically assigned to this group. Furthermore, given the limited number of LNs examined, the likelihood of underdiagnosis is significantly increased^10^.

As for the group with ≥25 LNs, in Table 5 we demonstrate that only the comparison between N-Ratio 3 and pN3 was statistically significant (11.0 vs. 30.0, p=0.021, p<0.05). Taking this into consideration, we can presume that the lack of statistical significance in the other comparisons may be attributed to the smaller sample size, as compared to N-Ratio 3 and pN3, and the superior prognostic ability of N-Ratio in predicting poorer outcomes.

It should be noted that at the Cancer Institute of the State of São Paulo, the center where all patients in this study were operated on, it is routine to perform extended lymphadenectomy, and it is hypothesized that the statistical values of N-Ratio would be even more significant for centers that operate with smaller lymph node resections.

The present study has some limitations. This is a retrospective research, in which we evaluated patients based on the experience of a single center. One of the main limitations is the lack of standardization in the cutoffs of the N-Ratio, which have been different in most studies in the literature, as well as the number of LNs that should be resected to obtain a viable and prognostic stage^21^. In the present study, we determined the N-Ratio categories based on the cutoff values determined by ROC curve. Furthermore, although some researchers state that the prognostic impact of the N-Ratio is restricted to patients with inadequate lymph node dissection, in this study it was not possible to assess the influence of the N-Ratio on cases with fewer than 15 LNs. In this case, although evaluating patients with fewer than 25 LNs, the number of patients was limited for some analyses. In fact, the mean number of lymph nodes in our study was 41.2, and D2 lymphadenectomy was performed in 85.6% of cases — which is higher than most studies. This may explain why we did not find significant differences between all N-Ratio categories when stratified by the number of LNs (25 LNs).

Possibly, if applied only in D1 cases, or in a larger cohort of patients with <25 LNs, some differences may be evidenced — which would possibly be those who would most benefit from prognostic determination by applying the N-Ratio.

## CONCLUSIONS

N-Ratio was an independent factor associated with survival in GC patients, being able to stratify especially those with more advanced lymph node disease (N-Ratio 3). As the N-Ratio does not weigh pN0 cases, an individualized prognosis index should be considered in those with a lymph node yield of less than 25.
